# An unusual case of acute kidney injury caused by obstructive uropathy revealing gastric cancer

**DOI:** 10.5339/qmj.2022.15

**Published:** 2022-03-04

**Authors:** Karima Boubaker, Mohamad Alkadi, Omar Fitouri, Ali Ibrahim Ali Rahil, Hassen Al Malki

**Affiliations:** ^1^Nephrology Department, Hamad Medical Corporation, Doha, Qatar E-mail: KBoubaker1@hamad.qa; ^2^Internal Medicine Department, Hamad Medical Corporation, Doha, Qatar

**Keywords:** Stomach neoplasms, acute kidney injury, ureteral obstruction

## Abstract

Acute kidney injury is a common complication in patients with cancer. Obstructive uropathy is a rare complication of gastric cancer and occurs mainly during treatment. Moreover, obstructive uropathy is rarely complicated by acute renal injury and thus requires emergency treatment. We report a rare case of anuric acute kidney injury that was actually obstructive uropathy secondary to gastric adenocarcinoma. Based on this case and literature review, malignancy should be considered in any patient not known to have cancer who presents with acute kidney injury caused by obstructive uropathy. This presentation reflects an advanced stage of malignancy. Non-enhanced computed tomography is valuable and should be the initial imaging study for diagnosing the obstruction and its cause. Early diagnosis and relief of the obstruction are associated with better recovery of the renal function.

## Introduction

Acute kidney injury is a common complication in patients with cancer, especially those with bladder, prostate, uterus, or cervix malignancies. It is associated with lower remission rates and increased mortality, hospital length of stay, and cost.^1–4^ Obstructive uropathy is a rare complication of gastric cancer and occurs mainly during treatment, and it is rarely complicated by acute kidney injury.^5^

Herein, we report a rare case of anuric acute kidney injury that was actually obstructive uropathy secondary to gastric adenocarcinoma.

## Case Report

**Figure 1.**
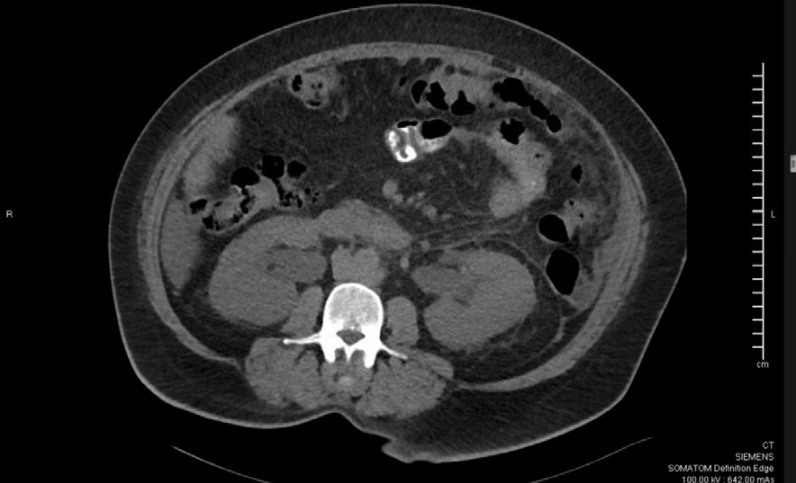
Computed tomography scan showing bilateral hydronephrosis

**Figure 2.**
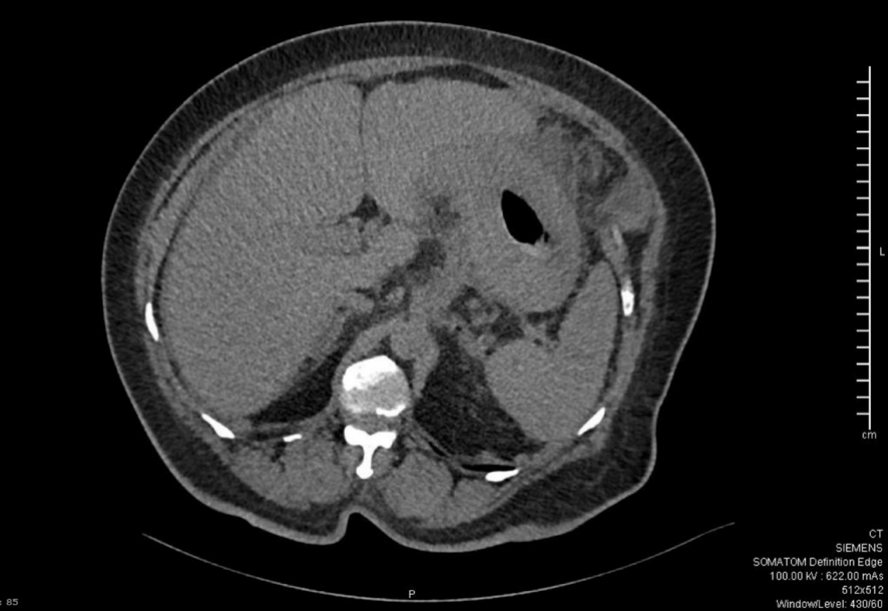
Computed tomography scan showing diffuse thickening of the stomach wall

**Figure 3.**
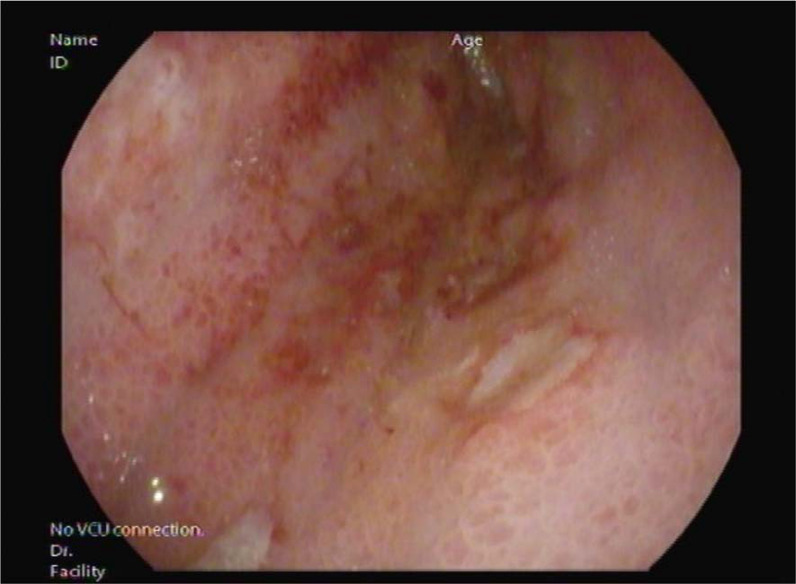
Esogastroduodenal endoscopy showing edematous and thickened stomach, with multiple ulcerations

**Figure 4.**
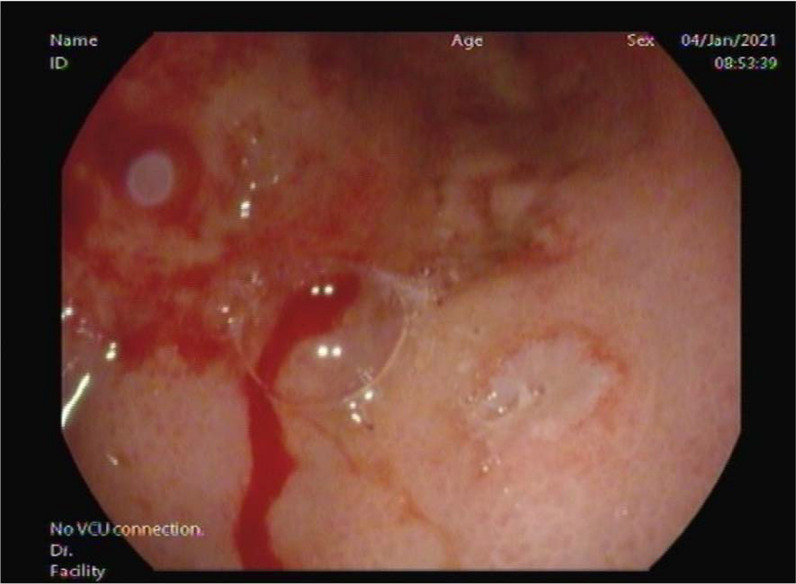
Esogastroduodenal endoscopy showing edematous and thickened stomach, with multiple ulcerations

A 59-year-old woman was admitted to the hospital for a sudden onset of anuria for 14 h. Before the onset, the patient had no known history of progressive decrease in urine output, recent color changes, or urinary habits. She reported an unintentional weight loss of 10 kg over the last 3 months associated with nausea, vomiting, decreased appetite, and mild periumbilical pain. She denied shortness of breath, peripheral limb swelling, or flank pain. She had a history of long-standing diabetes mellitus and hypertension. She visited the diabetic clinic until August 2018 and was then lost to follow-up. Her creatinine level was 57 μmol/L (normal range, 44–88 μmol/L), and her urine microalbumin-to-creatinine ratio was 6.9 mg/mL during her last clinic visit (normal range ≤ 3.5 mg/mL). Vital signs upon presentation were as follows: blood pressure, 193/106 mmHg; temperature, 36.8°C; heart rate, 98 beats per minute; respiratory rate, 18 breaths per minute; and oxygen saturation, 99% on room air. Her physical examination was unremarkable, with a non-tender, non-distended abdomen, and no lower limb edema was noted. A rectal examination was not performed. Laboratory results revealed severe renal impairment with a serum creatinine level of 657 μmol/L and urea level of 16.9 mmol/L. Other laboratory findings were as follows: sodium, 135 mmol/L; potassium, 4.9 mmol/L; chloride, 98 mmol/L; bicarbonate, 22 mmol/L; white blood cells, 6800; hemoglobin, 9.8 g/dL; corrected calcium, 2.61 mmol/L; and C-reactive protein, 18.5 mg/L. Abdominal and pelvic ultrasonography revealed normal right and left kidneys of 12.6 × 4.7 cm and 12.6 × 5.4 cm, respectively, with normal cortical echogenicity. Mild-to-moderate bilateral hydronephrosis was noted more on the right side with mild ascites. No perinephric collections were observed. The urinary bladder was empty at the time of examination. The uterus was bulky and heterogeneous, with no sizable adnexal lesions. Abdominal and pelvic computed tomography (CT) with oral contrast confirmed the mild bilateral hydronephrosis (Figure 1), but there were no renal or ureteric stones. There was diffuse thickening of the stomach wall with perigastric omental fat stranding, regional lymph node involvement, and mild free fluids in the abdomen and pelvis (Figure 2). There was thickening of the left pararenal, and the lateral conal fascia, liver, adrenals, spleen, and uterus appear unremarkable (Figure 2). The patient underwent bilateral JJ stent insertion, and her urine output increased immediately afterward. Her renal function also started improving, and her creatinine returned to baseline within 5 days. Esogastroduodenal endoscopy showed an edematous and thickened stomach, with multiple ulcerations and sluggish peristalsis, sparing the antral part (Figures 3 and 4). The scope passed easily into the duodenum, and no obstruction was seen. A gastric body biopsy showed poorly differentiated adenocarcinoma. The tests for human epidermal growth factor receptor 2 oncogene and *Helicobacter pylori* were negative. Serum levels of tumor markers carcinoembryonic antigen and carbohydrate antigen 19-9 were in the normal range. Endoscopic ultrasonography showed an indurated, diffuse gastric cancer with gastroesophageal junction extending to the body and angularis (linitis plastica). The lesion was hypoechoic and extended through the muscularis propria to the visceral serosa. There were more than 12 lymph nodes of different sizes. The staging evaluation included endoscopic ultrasonography and NM whole-body fluorodeoxyglucose-positron emission tomography/CT. The final global staging of the gastric adenocarcinoma was cT4a N3b M1, negative for *Helicobacter pylori*, and PDL score 10. The treatment of gastric cancer was palliative chemotherapy based on folinic acid, fluorouracil, and oxaliplatin, without fluorouracil bolus associated with nivolumab. After 2 months of follow-up, the patient had a creatinine level of 73 μmol/L, urea of 3.7 mmol/L, hemoglobin of 8.8 g/dL, and corrected calcium of 2.61 mmol/L.

## Discussion

Several teaching points can be made in this case. A peritoneal involvement of gastric adenocarcinoma can cause bilateral ureteral obstruction. Our patient had a rare presentation of gastric cancer with urinary tract obstruction causing acute kidney injury. Urinary tract obstruction is a rare cause of acute kidney injury in adults. However, it is a common cause of acute kidney injury in patients with cancer, especially those with malignancies of the bladder, prostate, uterus, or cervix, but exceptionally gastric cancer. Our patient presented with acute kidney injury and anuria with no significant electrolytes or acid–base imbalance and was not known to have neoplasia at presentation. Gastric cancer is one of the most common cancers worldwide.^6^ It may spread to the urinary tract by hematogenous or lymphatic channels or involve these structures by direct extension and likely compress rather than invade them.^7^ Signs and symptoms of metastatic disease to the urinary tract usually appear late in the course of gastric cancer and may only be recognized preterminal or at autopsy. Acute kidney injury caused by obstructive uropathy secondary to gastric cancer is rare. It occurred mainly during treatment of the cancerous malignancy^8–13^ but rarely at presentation (only in 17% of cases), as in our patient.^14^ Acute renal injury as a presentation of gastric cancer is mainly an obstructive injury rather than a renal injury caused by crescent glomerulonephritis.^15^ Acute kidney injury caused by obstructive uropathy is mainly due to intratubular obstruction of uric acid crystals in uric acid nephropathy, light chain casts in cast nephropathy, xanthine, hypoxanthine, calcium phosphate, or crystallization of certain drugs such as high-dose methotrexate.^16,17^ It is less likely secondary to extrinsic compression of the urinary tract from metastatic abdominal malignancies, which can result from peritoneal involvement, as in our patient,^14^ metastasis to the bladder,^18,19^ metastasis to the uterine cervix,^19^ or retroperitoneal fibrosis.^20^ Urinary tract obstruction secondary to gastric cancer can occur anywhere along the urinary tract, at one or multiple sites.^14^ It is symptomatic in half of the cases.^5^ Urinary tract obstruction is an occasional complication in patients with gastric cancer. It is rarely complicated by acute kidney injury, observed only in 8% of the cases, and due to bilateral obstruction in most patients as in our patient.^5,21^ Obstruction is less likely unilateral and occurs if the contralateral kidney is absent or has lesions such as acute tubular necrosis or in the presence of underlying kidney disease.^22^ Acute kidney injury is a result of decreased renal blood flow and glomerular filtration rate due to the activation of the renin–angiotensin system, prostaglandins, thromboxane, and kinin–kallikrein system.^23^ The clinicobiological spectrum of acute obstructive renal failure is a rise in renal function tests and acute urine retention, resulting in oligo anuria, as in our patient upon presentation. Acute urine retention can result in the inability to pass urine voluntarily. The patient can have abdominal or flank pain, frequently observed in 40% of the cases, and this was not observed in our patient.^5^ On physical examination, we can find a palpable bladder that is percussible and sensitive to pain when the patient is unable to pass urine or there was costovertebral angle tenderness. However, these symptoms are not always seen, as in our case. Estimating and measuring the glomerular filtration rate in patients with acute kidney injury are not recommended.^24^ An increase in cystatin C level demonstrates better performance compared with creatinine as a better indicator of the severity of obstructive uropathy.^25^ The diagnosis of urinary tract obstruction is usually established by imaging studies, which should be performed in all patients who present with acute kidney injury of unknown cause. They typically show hydronephrosis and can be characteristic of urinary tract involvement secondary to gastric cancer.^26^ Hydronephrosis is an anatomical diagnosis, which is characterized by the dilation of the urinary tract caused by gastric cancer-induced extrinsic compression of the ureter, culminating in urine retention that expands the upper urinary tract.^27^ It is likely bilateral, as in our patient.^5,21^ In some cases, it is unilateral, resulting in unilateral obstruction with an absent contralateral kidney, lesions such as acute tubular necrosis, or underlying kidney disease.^22^ Ultrasonography is the preferred imaging test for most patients with acute renal injury caused by obstructive uropathy, as it is safe, relatively inexpensive with a negative predictive value of 98%, and a positive predictive value reaching 70%,^28^ but it can be as low as 6% for mild hydronephrosis as an incidental finding.^28,29^ The dilation of the upper urinary tract appears as a hypoechoic area inside the renal cortex.^28^ Kidneys appear to be large.^30^ Visualizing the bladder is important when establishing hydronephrosis; if the urinary post-void volume exceeds 150 mL, it suggests urinary retention.^31^ Doppler ultrasonography can often detect the pulsatile movement of the urine into the bladder (ureteral jets) by B-mode sonography, and the absence or decreased frequency of these indicates urinary obstruction.^32^

In our patient, abdominal and pelvic ultrasonography revealed both kidneys with normal size and cortical echogenicity and bilateral hydronephrosis. The urinary bladder was empty at the time of examination. Non-contrast CT should be the initial study when an acute kidney injury caused by obstructive uropathy is suspected. A negative ultrasonography finding does not rule out the diagnosis. Urinary tract obstruction may occur with minimal hydronephrosis in early acute obstruction before the accumulation of urine occurs or when urine production is reduced for other reasons, such as parenchymal renal disease, or in the setting of retroperitoneal fibrosis.^33,34^ A grading system has been applied to determine the severity of hydronephrosis from the minimal separation of the central sinus fat by fluid to the discontinuity of the sinus fat.^35^ CT is also useful for recognizing pelvic and abdominal masses. In our patient, a CT scan showed mild bilateral hydronephrosis. The ureter stricture has a typically ring-like appearance.^26^ CT reveals thickening of both gastric and renal pelvic walls with the infiltration of the renal sinus fat, as in our case,^26^ obvious para-aortic lymph node, and/or pelvic metastases.^14^ Typically, intravenous urogram shows thread-like ureteral stricture.^26^ Magnetic resonance imaging (MRI) without gadolinium enhancement may be an alternative to CT,^36,37^ as it does not expose patients to radiation and has a diagnostic yield similar to CT. The administration of gadolinium during MRI has been strongly linked to an often-severe disease called nephrogenic systemic fibrosis in patients with severe renal impairment.^38^ Consequently, gadolinium-based imaging is recommended to be safely used after recovery from acute kidney injury. Pathological examination of gastric cancer frequently shows undifferentiated and poorly differentiated adenocarcinoma in 70%–76%, as in our patient,^14,26^ followed by the differentiated type in 12%^26^ and the unknown histological type in 8% of the cases.^29^ Endoscopic ultrasonography is performed for local staging of gastric adenocarcinoma. Pathologically, the undifferentiated type of gastric cancer tends to spread, infiltrating the vessels, nerves, and lymphatics along the course without alteration of the ordinary anatomical structures. In such cases, the mucosal surface of the urinary tract tended to be spared despite extensive tumor invasion.^26^ However, in the present case, the lesion was extending through the muscularis propria to the visceral serosa. The presence of acute kidney injury caused by obstructive uropathy during gastric cancer usually indicates an advanced stage of malignancy with metastasis generally associated with an extremely poor prognosis.^21^ Vital and renal prognosis is the worst in male older patients, with a death probability three times higher compared to other patients.^39^ At this stage, the treatment of gastric cancer is only palliative, based on chemotherapy and immunotherapy. Our patient received palliative chemotherapy. Acute kidney injury caused by obstructive uropathy required an urgent palliative urinary diversion. The aim is to relieve the urinary tract obstruction by percutaneous nephrostomy or cystoscopic placement of a ureteral bilateral JJ stent,^14^ which alleviates the expansion of the tract and urine accumulation and reverts acute kidney injury.^40^ Our patient underwent early ureteral bilateral JJ stent insertion with a total recovery of renal function within 5 days without immediate complications. Percutaneous nephrostomy was preferred in one study because of the high failure rate of JJ stents.^14^ Although it is a well-established interventional method, it is associated with significant morbidity, thus affecting the quality of life.^40^ Recovery of baseline renal function is usually seen in the first 7–10 days after relief of the obstruction^42,43^ and will depend on the severity and duration of the obstruction, as well as other potential complicating factors, such as hypertension, infection, or pre-existing renal disease.^44^ In our patient, renal function started to improve after the procedure, with the normalization of the creatinine level and an in increase in urine output despite comorbidities. Electrolyte disturbances and hypovolemia should be avoided in cases of polyuria occurring after the urinary obstruction was cleared. Post-obstructive diuresis is a result of the elevated compression of the urinary tract, which compromises the capacity of renal tubules to concentrate urine and the high amounts of urea in renal tubules, which cause osmotic diuresis.^45,46^ Intravenous fluids and electrolyte replacement should be given to patients with polyuria that compromises hemodynamics.^47^ Renal replacement therapy can be indicated at presentation in case of fluid overload, urgent hydro-electrolytic alterations, imbalance in acid–base hemostasis or, rarely, when the relief of obstruction is insufficient to mitigate acute kidney injury-associated complications.^47^ The probability of requiring renal replacement therapy increases in cases with a history of chronic kidney disease.^47^ Our patient, who has a previously normal renal function, did not require renal replacement therapy at presentation and after bilateral JJ stent insertion. Currently, there is no medical treatment for obstructive nephropathy. Still, some medications have been tried to limit the progression of acute kidney injury to chronic kidney disease, including hydrogen sulfide, which was proven to hinder fibrosis^48^ and angiotensin-converting enzyme inhibitors.^23^ The prognosis is extremely poor. Survival is improved by both chemotherapy and immunotherapy using immune checkpoint and was the only independent predictor of survival.^5,49^ The median survival is 3.1–5.8 months in patients who did not receive and 11.2 months in those who received chemotherapy.^5^

## Conclusions

We presented a rare cause of acute kidney injury due to bilateral ureteral obstruction caused by a peritoneal involvement of gastric adenocarcinoma. Based on this case and literature review, malignancy should be considered in any patient not known to have cancer who presents with acute kidney injury caused by obstructive uropathy. This presentation reflects an advanced stage of malignancy. CT without contrast is valuable and should be the initial imaging study to diagnose the obstruction and its cause. Early diagnosis and relief of obstruction are associated with better recovery of renal function.

